# Rasori

**Published:** 1838-07

**Authors:** 


### RASORI.

Giovanni Rasori was born at Parma, in 1766. After an enlarged and varied education, both at Italy and in foreign countries, including England, where he remained a considerable time, he was appointed professor of medicine, first in the university of Pavia, and afterwards at Pisa. He went from this to Milan, where, in the year 1799, he published his first work, "Analisi del preteso genio d'Ippocrate." He was in Genoa during the siege of that city; and, in 1801, published his celebrated account of the fever which prevailed there, and in the treatment of which he shewed so much zeal and obtained so much success. This is his greatest work, and that on which his fame chiefly rests, as it was in this that he first announced the new practice of giving emetic tartar in large doses, which has since become so famous. It is entitled "Storia della Febbre epidemica di Genova, degli anni 1799 e 1800." After the conquest of Italy by Napoleon, he returned to Milan, and soon acquired great reputation, both as a practitioner and clinical teacher. Rasori continued to enjoy great reputation and consideration at Milan during the reign of Napoleon; but, upon the return of Lombardy to the Austrian dominion, he was dispossessed of his appointments, and imprisoned for several years. On being restored to liberty, he resumed his practice and his clinical instruction at Milan, where he died in April, 1837. In the earlier part of his life he translated into Italian Brown's "Elements of Medicine" and Darwin's "Zoonomia." In 1830, he published two volumes on clinical medicine, under the title "Opuscoli di Medicina Clinica;" and, at the time of his death, he was engaged in printing what he regarded as his greatest work, and in the preparation of which (he tells us in his preface,) he had been engaged the greater part of his life. This has since been published in two volumes, under the title "Teoria della Flogosi," of which we hope to give an account in an early Number.

It is well known that it is to Rasori that we are indebted for the introduction into general practice of emetic tartar in large doses, as a remedy in febrile and inflammatory diseases, although it had been previously employed, by Dr. Marryat in this country, in similar doses; and we are of opinion that his name will be handed down to posterity, more by this circumstance than by his writings. Rasori was greatly esteemed at Milan, where a statue is about to be erected to his memory at the public expense.

---

